# Complications related to use of mesh implants in surgical treatment of stress urinary incontinence and pelvic organ prolapse: infection or inflammation?

**DOI:** 10.1007/s00345-019-02679-w

**Published:** 2019-02-13

**Authors:** Naşide Mangir, Sabiniano Roman, Christopher R. Chapple, Sheila MacNeil

**Affiliations:** 1 Department of Material Science and Engineering, Kroto Research Institute, University of Sheffield, Sheffield, UK; 2 Department of Urology, Royal Hallamshire Hospital, Sheffield, UK

**Keywords:** Stress urinary incontinence, Pelvic organ prolapse, Surgical mesh, Polypropylene, Infection, Inflammation

## Abstract

The surgical mesh material used in the surgical treatment of stress urinary incontinence (SUI) and pelvic organ prolapse (POP) in women is associated with significant complications in some women. This has recently become a public health issue with involvement of national parliaments and regulatory bodies. The occurrence of mesh complications is thought to be a result of multifactorial processes involving problems related to the material design, the surgical techniques used and disease, and patient-related factors. However, the infectious complications and mesh–tissue interactions are least studied. The aim of this article is to review any previous clinical and basic scientific evidence about the contribution of infectious and inflammatory processes to the occurrence of mesh-related complications in SUI and POP. A literature search for the relevant publications without any time limits was performed on the Medline database. There is evidence to show that vaginal meshes are associated with an unfavourable host response at the site of implantation. The underlying mechanisms leading to this type of host response is not completely clear. Mesh contamination with vaginal flora during surgical implantation can be a factor modifying the host response if there is a subclinical infection that can trigger a sustained inflammation. More basic science research is required to identify the biological mechanisms causing a sustained inflammation at the mesh–tissue interface that can then lead to contraction, mesh erosion, and pain.

## Introduction

Surgical mesh, mostly made of polypropylene (PPL), used in the surgical treatment of stress urinary incontinence (SUI) and pelvic organ prolapse (POP) has become a public health problem being frequently quoted as the biggest scandal after the thalidomide disaster. Recently, New Zealand became the first country to ban these products for use in transvaginal POP repair [1]. In the UK, two parliaments undertook public enquiries as a result of immense pressure from patient organizations leading to a recent suspension of vaginal mesh products for both SUI and POP [2]. On the other hand, the British Society of Urogynaecologists expressed strong disagreement with the decision stating that many women with SUI would be deprived of an effective and safe treatment modality as a result of this decision, while they agreed with the decision to cease using vaginal mesh for POP [3]. All these discussions are being widely covered in the media with a lot of public attention.

The success and complication rates for vaginal mesh surgeries appear to be highly variable with better risk/benefit ratios when used for treatment of SUI as compared to POP. This implies that factors related to disease mechanisms, surgical techniques, and material properties also have an impact on the occurrence of vaginal mesh complications. One important factor is probably related to material properties of the surgical mesh and its suitability when used in the pelvic floor. The surgical mesh became available for surgical use shortly after the plastics revolution and it was a significant advancement in materials science at the time that it was introduced [4]. The plastic materials provided significant advantages over the metal prosthesis in soft-tissue reconstruction with their ductility and strength. However, plastic meshes were not as resistant to infection as the metals that necessitated modifications to material properties to decrease bacterial attachment and adaptations of the surgical technique of hernia repair to prevent long-term colonization of the mesh [5].

Mesh-related infections are relatively rare after mesh augmented pelvic floor repair procedures; however, when they do occur, they can significantly compromise patients’ well-being and can lead to excision of the mesh implant. This year, we sadly learnt from a newspaper article that a well-known mesh campaigner died of sepsis attributed to recurrent urinary tract infections (UTIs) developed after a mid-urethral sling implantation to treat SUI [6]. Mesh-related complications due to infection have been reported to occur in 0–8% of all cases, but generally speaking, they appear to be less than 1% in transvaginal mesh implantations for the treatment of both SUI and POP [7, 8]. These figures reflect clinically evident infection with typical systemic findings (fever, malaise, etc.) and local signs of infection at the site of implantation. Mesh infection is also thought to be asymptomatic (silent), but it can actually interfere with the successful integration of the mesh into host tissues leading to mesh exposure in some cases [9]. A positive bacterial culture was obtained from 77% of the vaginal meshes explanted due to pain, mesh erosion, mesh infection, and recurrent UTIs [10]. Therefore, mesh-related infections can be a solitary complication of vaginal mesh surgeries, and at the same time, it can be one of the factors in a multifactorial process underlying other mesh-related complications such as exposure and pain.

Infectious complications have also been a major issue for other polymer-based biomedical implants designed for soft-tissue replacement/augmentation such as the breast implants. The relationship between the biomaterial infection and implant-related complications is relatively well studied in this context. For implant-based breast augmentation surgeries, capsular contraction is known to be a major cause of reoperation [11]. Theories on the occurrence of capsular contraction are hypertrophic scar, myofibroblast activation, silicone gel leak, haematoma, and infection. The latter suggests that a microbial colonization of the implant causes a persistent low-grade infection leading to what is called a subclinical infection and capsular contraction. Breast implants explanted for capsular contracture revealed a 41% culture positivity for microorganisms which were mainly skin flora [12]. Although this theory could explain some of these clinical complications, clinical relevance is limited due to lack of non-invasive methods that can provide such evidence.

The aim of this article is to review available clinical and basic scientific evidence about infection complications of vaginal mesh surgeries. We first investigated the specific tissue reactions to the implanted vaginal meshes. We then considered vaginal mesh colonization and infection as mechanisms to explain mesh-related complications in urogynecological surgery.

## Methods

A Medline search was conducted on October 2018 using the following as subject headings, keywords, and text words: (stress urinary incontinence OR pelvic organ prolapse) AND (surgical mesh OR polypropylene) AND (infection OR wound infection OR post-operative complications OR intraoperative complications). No time limits were applied to the search. A total of 168 abstracts were retrieved. All relevant articles were included. In addition, reference lists of selected manuscripts were checked manually for eligible articles.

### Infectious complications of vaginal mesh surgeries in clinical practice

Vaginal mesh is used in urogyneacological surgeries mainly to treat SUI and POP. It is used in the female pelvic floor in three main ways: transvaginal treatment for SUI, transabdominal repair of POP, and transvaginal repair of POP.

For transabdominal implantations, namely, abdominal sacrocolpo(histero)pexy operations, the mesh material is used to attach the apex of the vagina or uterus to the sacrum, replacing defective or weak cardinal-uterosacral ligaments constituting level I support structures [13]. These ligaments are thick and strong collagenous fibres extending both vertically and posteriorly towards the sacrum, meaning that it is not necessarily flexible, but strong in the vertical direction which matches with the mechanical properties of the surgical mesh. In addition, in these operations, the mesh does not traverse a clean contaminated surgical field and it does not lie in close proximation to skin. Thus, the mesh in abdominal implantations is biomechanically more fit for purpose for this application and the chances of contamination during implantation is less compared to vaginal implantations. The success of transabdominal repairs is very good at 97–100% [14], although mesh erosion still occurring in up to 6% by 2 years [15] and 10% in 7 year follow-up [16]. Mesh infection rates are also thought to occur less in abdominal POP repairs compared to vaginal POP repairs, since the first approach avoids contamination of the mesh during insertion [17]. Furthermore, avoiding a hysterectomy during abdominal sacrocolpopexies is recommended to reduce likelihood of mesh complications by preventing the contact of the mesh with vaginal microbial flora.

In transvaginal POP repair procedures, the vaginal support structures, mainly at level II, are attached to stronger ligaments in the pelvic floor (e.g., sacrospinous or uterosacral ligaments) or are augmented with a suture repair in pubocervical or rectovaginal fascia (anterior and posterior colporrhaphy procedures). This fascia is mainly composed of smooth muscle and collagen/elastin, which are the active biomechanical components of the pelvic floor that are probably subjected to not well-defined multidimensional forces. More importantly, during these operations, the mesh traverses a clean contaminated surgical field which increases the chances of contamination. In addition, transvaginal repairs are essentially mesh ‘onlay’ procedures, particularly anterior and posterior colporraphy procedures, which make them prone to colonization by vaginal microbial flora, as they lie very close to the skin [18].

The transvaginal mesh tape insertions for SUI are slightly different than other transvaginal mesh insertions, because in these operations, a smaller surface area of the mesh lies in close proximation to the vaginal skin, but maybe more importantly, the theoretical basis for use of the mesh for SUI is better studied with better defined targets for surgical treatment. For example, placement of a synthetic tape underneath the mid urethra was conceptualized with the introduction of mid-urethral sling surgeries with demonstration of pre- and post-operative urethral pressures.

Hence, although the transvaginal route has been the most commonly used route for POP repair, the safety of mesh augmented transvaginal POP repair procedures is now widely questioned with a mesh erosion rate of 8% in 1–3 year follow-up and which can go up to 42% in longer term follow-up [19]. There appears to be a consensus on lack of safety with transvaginal mesh implantation for POP. In contrast, currently, tension-free vaginal tape procedures for SUI have long-term subjective cure rates of up to 93% [20] with mesh-related complications occurring in 4% of patients [21]. Current expert opinion suggests that the benefits of these operations still outweigh the risks with a high level of evidence.

With regard to infectious complications of transvaginal mesh surgeries, the most recent PROSPECT trial [22] demonstrated that the rate of infectious complications with the vaginal mesh was less than 1% [22], although higher rates have been reported of up to 8% [9]. However, in a series of mesh explantation surgeries after treatment of SUI and POP, mesh exposure without signs of infection was responsible for 44 of 83 cases, with 30 of 84 meshes excised due to infection [23].

### Tissue reactions to the surgical mesh and the scientific evidence for mesh infection

Occurrence of vaginal mesh-related complications, as we see in the daily clinical practice, are probably multifactorial including the inherent complexity of pelvic floor disorders that are still not incompletely understood [24], the material and biomechanical properties of the mesh being unsuitable for use in pelvic floor, limitations pertinent to the surgical techniques used, and failure of regulatory processes for approval and surveillance of implantable medical devices.

Infectious complications of the vaginal mesh can be thought of as a clinical entity with specific signs and positive culture results, but also it can be a subclinical infection affecting the normal host response to the mesh and its’ tissue integration. Alternatively, we can observe complications associated with an inflammatory reaction to the mesh material with a completely sterile mesh without infection. In this section, we will review the available evidence on the host tissue response to the PPL mesh and how this could relate to clinical outcomes.

#### Host response to PPL mesh

Surgical mesh became available as a material after the plastics revolution and started to be used in hernia repair [4]. Plastic materials provided significant advantages over the metal prosthesis, the only available alternative then used in soft-tissue reconstruction because of their better ductility and strength. Plastics, however, came with a new set of material properties that was initially problematic when used with traditional material design strategies and available surgical techniques of implantation. Some of these properties needed to be optimised over the years to obtain the best treatment outcomes [5]. These improvements were made in the context of hernia surgeries over 50 years before mesh was used in pelvic floor repair.

The biocompatibility of the mesh is mainly determined by its textile properties, namely, the porosity and the pore size. Lighter weight meshes with large pores are known to integrate better into host tissues with less foreign body reaction, fibrosis, and the associated pain sensation [25, 26]. Clinical studies comparing heavy and light-weight PPL mesh materials implanted for inguinal hernia repairs demonstrated less pain and less sensation of a foreign material with lighter meshes [27]. However, lighter weight meshes are more flexible which caused effective loss of pores after mechanical loading in vivo and this led to some issues for the definition of pore size and pore stability [28]. Prolapse meshes are thought to be more likely to lose their pores after implantation in vivo compared to hernia meshes Auxetic materials have been developed for use in prolapse repair [29]; however, their efficacy in reducing mesh-related complications is yet to be explored.

It has also been demonstrated repeatedly that the type of the mesh material affects its biocompatibility. Meshes made of polyester or polytetrafluoroethylene (PTFE) are known to be more susceptible to bacterial colonization, and efforts have been focused on the improvement of their antibacterial properties. For example, PTFE has been modified to release two antimicrobial molecules (silver salts and chlorhexidine) used in vaginal implantations in a small series [30]. However, it is widely accepted today that implantations through the vaginal route increase the risk of contamination and that the best material is monofilament macroporous PPL for this application [31].

In case of the PPL mesh, the host response has traditionally been studied for applications in abdominal hernia repair with recent evidence focused on vaginal implantations. The PPL mesh is known to trigger an inflammatory response characterized by polarization of macrophages towards an M1 phenotype, as opposed to M2. An M1 phenotype leads to a pro-inflammatory response, while an M2 results in a constructive remodelling response [32]. In addition, the M1/M2 ratio has been shown to be less favourable with increased molecular weight PP mesh and with smaller pore sizes, suggesting that the mesh burden (the amount of mesh in contact with tissues) is a factor influencing its biocompatibility [33].

Biocompatibility is defined for each specific application of a biomaterial as its ability to perform with an appropriate host response [34]. The biocompatibility of the PPL mesh for applications in the pelvic floor started to being defined after 2007 in the sheep [35]. The sheep have a vagina that is similar in size to the human vagina allowing larger pieces of the mesh to be implanted and they can spontaneously develop POP. A site-specific host response to PPL mesh in sheep models has demonstrated mesh-related complications (exposure and contraction) to occur significantly more in transvaginal mesh implantations as compared to abdominal implantations, where the same materials caused less than 10% contraction and no erosion [36]. Later on, clinical data from women who underwent vaginal mesh excision due to complications revealed an M1 (pro-inflammatory) macrophage response even years after the implantation of mesh, with a higher expression of proteolytic enzymes in explants of women who had mesh exposure compared to women with pain [37].

#### PPL-related infection

The events in the tissue–material interface leading to device-related infections are well studied. Initially, the microorganisms attach and adhere to the surface of the material via physicochemical interactions including Van der Waals forces, hydrophobic, and electrical interactions. Microorganisms can also attach on to the proteins adsorbed on the surface of the material. After attachment, microorganisms proliferate and form multi-layered clusters via specific intercellular adhesion polysaccharides [38]. The presence of such accumulated biofilms has been demonstrated on several implanted devices including these surgical meshes [39].

The presence of a mesh-related infection can modify the host tissue response to the implanted material [40]. As soon as a biomaterial is implanted, a ‘race to surface’ begins between the host cells and the microorganisms. A biomaterial-associated infection will affect the integration of the implant into the host. Although it is easy to distinguish between an implant which is clinically infected and a successfully integrated implant, it is not so easy to detect low levels of infection in an implant. Furthermore, this situation is a dynamic process that can change over years.

Bacteria generally form biofilms on the surface of biomedical implants. Biofilms are aggregates of bacteria with a surrounding extracellular matrix (extracellular polymeric substances) that is tightly attached to the biomaterial surface. Bacteria in biofilms are resistant to antimicrobial therapies and they can easily evade the host immune responses giving rise to a state of chronic inflammation [41]. The relationship between microbial biofilms and capsular contraction with breast implants has been extensively studied in pre-clinical and clinical studies, which is reviewed elsewhere [42]. It appears that biofilm formation is an acknowledged factor increasing the occurrence of capsular contraction. Although the mechanisms underlying mesh contraction by the host tissues are not clear, it can be argued that bacterial colonization of the vaginal mesh can affect the host response against the mesh and can contribute to mesh contraction in the absence of obvious signs of infection.

At the time of writing, there have not been enough studies reported to support or refute this hypothesis. Histological analysis of 100-explanted meshes revealed a periprosthetic tissue reaction identical to that of a periprosthetic abscess, regardless of an infectious cause of mesh explantation, and/or a chronic inflammation rich in giant cells and mononuclear cells [43]. However, experimental studies in rats have demonstrated that both absorbable and non-absorbable meshes shrink more when they are infected [44].

In conclusion, for any given synthetic implant, there will be a host response. The ideal situation is that the biomaterial and the host tissues can find a state of mutually acceptable co-existence.

#### The relationship between surgical technique and infection

SUI and POP commonly occur together due to challenges which the female pelvic floor must cope with. A combination of genetic and acquired factors that are most probably aggravated by childbirth lead to the occurrence of SUI and POP. Although the exact mechanisms by which an interaction of these factors results in pelvic floor disorders are not completely elucidated, the clinical picture involves initial mechanical damage to the pelvic floor that generally follows birth trauma, previous pelvic surgeries, menopause, and increasing age. Current surgical treatments for SUI and POP are based on restoration of normal anatomy in the female pelvic floor either augmented by mesh or not.

PPL mesh was first designed for use in the treatment of abdominal hernia. For its use in this application, the material properties of the mesh and the surgical technique of implantation developed hand by hand over years to obtain best outcomes for hernia repairs. For example, in incisional hernia repair, onlay mesh repairs were replaced with sublay repairs, where the mesh is placed underneath a thick muscle tissue (retro-rectus) in a well-vascularized wound bed and away from the skin. Onlay mesh repairs required a large area of the mesh to stay in very close proximity to skin increasing the chances of mesh colonization and infection [45].

When adopting the mesh for vaginal mesh implantations, the design requirements for specific application in the pelvic floor were not considered. In vaginal mesh implantations, the mesh stays in very close proximation to the vaginal mucosa, as there are no natural tissue planes in this region such as subcutaneous or muscle tissue layers, unlike in abdominal implantations, where the mesh material is implanted in-between clearly identifiable fat, muscle, and fascia tissue planes.

Furthermore, there are other observations supporting the argument that an inflammatory reaction to the mesh or a subclinical infection caused by the introduction of the mesh may contribute to the occurrence of mesh-related complications. It has been repeatedly demonstrated that vaginal mesh complications are known to increase with increasing amounts of mesh used [36, 46]. In addition, clinical studies showed that avoiding an overlapping suture line during mesh implantation reduces mesh exposure [47, 48]. In addition, mesh exposure mostly occurs in the midline, suggesting a poor wound healing affected by the presence of the mesh [49]. Taken all together, this implies that mesh erosion can be followed from an abnormal wound healing of the incised vaginal mucosa due to a poorly vascularized wound bed combined with the surgical intervention and the presence of large amount of mesh material.

### Pathogens detected in infected vaginal mesh

For abdominal hernia meshes, *Staphylococcus aureus* (S aureus) is the most commonly isolated organism (more than 80% of cases) followed by *E. coli, Enterococcus,* and *Candida* [50]. Of the isolated S aureus, most were methicillin-resistant S aureus. Microbiologic analysis of explanted vaginal meshes demonstrated multi-microbial cultures in the majority of the cases (31%), and when solitary bacteria grew, coagulase-negative Staphylococci, *E. coli*, Proteus mirabilis, and Streptococcus agalactiae were detected, the quantity of which mostly is less than 10^3^/mL [51]. In an analysis of 175 excised mesh specimens, 77% of cultures were positive with 37% being positive for at least one pathogenic bacteria. Staphylococcus was the most commonly isolated organism followed by *Enterococcus* and *Finegoldia magna* [10].

### Methods to prevent infectious complications after vaginal mesh implantation

For any implantable or indwelling medical device, infection in the acute or chronic setting is an issue. When an infection related to a medical device occurs, the clinician needs to make a judgement whether to salvage the implanted device or to remove the infected device. This clinical decision will need to take into account several key factors including the importance of the device to the patients’ survival/wellbeing and the ease of removal/replicability of the device, efficacy of the antibiotic therapy used, and the factors related to patients’ clinical situation such as existence of immunosuppression or sepsis. Often, the removal of the device is required, because an antibiotic/antimicrobial therapy alone is generally not sufficient to clear the infection due to biofilm formation.

Strategies to prevent mesh-related infections start with recognition of factors that increase the likelihood of vaginal mesh infections. Several pre-operative patient-related risk factors have been suggested to influence the mesh-related complications including smoking, age, obesity, diabetes, immunocompromised status, and vaginal atrophy. Although specific risk factors that increase mesh infection have not been identified, smoking and obesity are risk factors for vaginal mesh exposure [31]. This issue has been studied more for the abdominal hernia repair procedures, suggesting that patient-related factors such as smoking, uncontrolled diabetes, obesity, and previous hernia repair can increase the risk of infectious complications with mesh. In addition, risk prediction tools are available for use with individual patients undergoing abdominal hernia repair [50]. Therefore, optimization of pre-operative risk factors can be a first step in prevention of infections.

Specific guidance is not available on pre-operative measures to reduce mesh infections. Standard infection control measures including hand hygiene, cutaneous asepsis, and prophylactic antibiotics can be considered. The only available guidance has been published by the French college of Obstetrics and Gynecology on prevention of complications related to use of prosthetic meshes in prolapse surgery [31]. Use of antibiotic prophylaxis, cleaning with an antiseptic foam solution followed by disinfection of the surgical site, use of double gloves and change of gloves at each stage of the operation, removing the package of the mesh at the very last moment, and manipulation of the mesh with a clean pair of gloves are all recommended. In addition, minimizing the surface area of the implanted mesh, good haemostasis before closure, and intermittent irrigation of the hernia sac/implant wound bed with antibiotic solution have also been described for hernia cases; however, the relevance of these on vaginal mesh procedures is not known.

#### Novel approaches in materials’ design to reduce mesh-related infections

Material properties of the surgical mesh can be modified to make it more resistant to colonization with bacteria. Surface modification and antimicrobial functionalisation of the biomedical implants have been the most frequently studied strategies. In the context of hernia meshes, soaking the mesh in an antimicrobial solution and coating the mesh with drug releasing polymers or antimicrobial metals such as silver nanoparticles have been studied.

In pelvic floor meshes, coating the PPL mesh with silver nanoparticles was found to decrease bacterial attachment in vivo [52]. Because the nondegradable meshes are not good drug releasing polymers, and only soaking strategies fail to achieve a sustained release state, coating the mesh with other polymers such as polylactic acid has been used as a strategy for carrying the antimicrobials [53]. Since there are limitations of this drug releasing approaches to achieve long-term release of the antimicrobials, other ways of incorporating agents into the mesh material have also been studied. Chemical modification of polymer surfaces to bind antimicrobial agents has also been studied experimentally [54].

Recent issues on the use of vaginal PPL mesh and the search for alternative materials have led to emergence of degradable or non-degradable electrospun materials as alternatives [55, 56]. In animal experiments, these materials have been repeatedly demonstrated to be infiltrated by host tissues soon after implantation and the host response to these materials has been characterized as driven by an M2 (remodelling) response [57]. A commonly cited concerns about these materials are their pore sizes in relation with their ability to accommodate host tissue cells before the pores invaded by the microorganisms.

The mechanisms underlying microbial attachment to electrospun surfaces are not extensively studied. The attachment of bacteria to the fibres of the electrospun materials is probably different than those on the flat surfaces that are relatively better described [58]. The nanotopography, chemistry, and roughness of the micro/nanofibres of the electropsun matrices can theoretically affect the attachment of bacteria. It was demonstrated that the fibre diameter influenced attachment of bacteria to the fibre, with smaller diameters decreasing attachment in polystyrene electrospun mesh [59]. Fibre diameter also influences the ability of the bacteria to proliferate and colonize the scaffold.

The attachment of cells to electropun fibres is known to be affected by the ultrastructural arrangement of the fibres within the electrospun mesh such as inter-fibre distance (pore size) and fibre alignment [60]. The attachment of bacteria on to these surfaces can also be expected to be influenced by the same ultrastructural properties. Furthermore, functionalization of the electrospun scaffolds with drugs or surface modifications can have effects on bacterial attachment that needs to be considered. Ideally, these surfaces would be designed, so that they facilitate attachment of host cells while inhibiting bacterial attachment, proliferation, and biofilm formation.

## Conclusion

In summary, at the time of writing, PPL mesh implants are known to cause an unfavourable host response with persistent inflammation leading to the clinical observed mesh-related complications. Some clinical and scientific evidence suggests that a subclinical infection due to a contaminated mesh can contribute to a sustained inflammation at the mesh-tissue interface leading to a poor tissue integration; nevertheless, to verify this, more research is required to identify the specific underlying mechanisms. Finally, methods to prevent infection are described.
